# Identification of individuals at high risk of developing rheumatoid arthritis: a balanced random forest model in a cohort of 1544 first-degree relatives

**DOI:** 10.1136/rmdopen-2025-005773

**Published:** 2025-11-27

**Authors:** Romain Aymon, Céline Lamacchia, Benoit Thomas P Gilbert, Maresa Grundhuber, Isabel Gehring, Sascha Swiniarski, Olivia Studer, Zubeyir Salis, Romain Guemara, David Spoerl, Jean Dudler, Burkhard Möller, Diana Dan, Laure Brulhart, Ines Von Mühlenen, Diego Kyburz, Andrea Rubbert-Roth, Adrian Ciurea, Ruediger Mueller, Delphine S Courvoisier, Axel Finckh

**Affiliations:** 1 Division of Rheumatology, Geneva University Hospitals, Geneve, Switzerland; 2 Thermo Fisher Scientific, Freiburg, Germany; 3 Université de Montpellier, Montpellier, France; 4 Physiology and Experimental Medicine of Heart and Muscles, Montpellier, France; 5 Department of Immunology and Allergy, Geneva University Hospitals, Geneve, Switzerland; 6 Department of Rheumatology, HFR, Fribourg, Switzerland; 7 Rheumatology and Immunology, Inselspital Universitatsspital Bern, Bern, BE, Switzerland; 8 Rheumatology, Lausanne University Hospital, Lausanne, Switzerland; 9 Faculty of Medicine, University of Lausanne, Lausanne, Switzerland; 10 Rheumatology, Réseau hospitalier neuchâtelois, La Chaux-de-Fonds, Switzerland; 11 Rheumatology Office, Rheuma-Basel, Basel, Switzerland; 12 Department of Rheumatology, University Hospital Basel, Basel, Switzerland; 13 Department of Biomedicine, University of Basel, Basel, BS, Switzerland; 14 Division of Rheumatology and Immunology, Kantonsspital St Gallen, Sankt Gallen, SG, Switzerland; 15 Department of Rheumatology, University of Zurich, University Hospital Zurich, Zurich, Switzerland; 16 Rheumazentrum Ostschweiz, St. Gallen, Switzerland; 17 Geneva Centre for Inflammation Research, Geneva University Hospitals, Geneve, Switzerland

**Keywords:** Arthritis, Rheumatoid, Biomarkers, Epidemiology, Machine Learning, Sensitivity and Specificity

## Abstract

**Objectives:**

To identify in a genetically susceptible population individuals at higher risk of developing rheumatoid arthritis (RA) using a classification approach combining known epidemiological risk factors, serological biomarkers, genetics, clinical signs and symptoms.

**Methods:**

We used data from the prospective SCREEN-RA (Evaluation of a SCREENing strategy for Rheumatoid Arthritis) cohort of 1540 first-degree relatives of RA patients (RA-FDRs). The primary outcome was the development of RA. Additionally, we used seropositive inflammatory arthritis (IA) as a secondary outcome for exploratory analyses. Balanced random forest (BRF) models were fit and evaluated through fivefold cross-validation to avoid overfitting. We chose a classification threshold that targeted high sensitivity.

**Results:**

After a mean follow-up of 7.1 years, 27 participants developed RA and 126 developed seropositive IA. The BRF demonstrated moderate predictive performance, characterised by high sensitivity (≥0.85) but modest specificity. Rheumatoid factors (RFs) had the highest importance in RA prediction, followed by symptoms of ‘clinically suspected arthralgia’ (CSA) scale. Age, gender and anti-RA33 autoantibodies were the main variables for the prediction of seropositive IA.

**Conclusions:**

Overall, the results demonstrate that predicting RA by combining genetics, serological biomarkers, epidemiological risk factors and clinical signs is promising, although model generalisation remains challenging. The low prevalence of RA in the cohort complicates the development of highly accurate prediction models. Future efforts should focus on including external validation and potentially incorporating additional biomarkers to enhance the sensitivity and overall performance of the predictive tests.

**WHAT IS ALREADY KNOWN ON THIS TOPIC**

- It is currently assumed that a primary or secondary intervention during the preclinical phases of rheumatoid arthritis (RA), particularly in high-risk individuals, may allow to prevent the disease altogether.
- However, the current prediction models and strategies to identify ‘at-risk’ individuals for preventive intervention are still to be improved.

**WHAT THIS STUDY ADDS**

- The present study includes a wide array of risk factors (epidemiological, serological, genetics and clinical symptoms) in a cohort of first-degree relatives of RA patients.
- By using a balanced random forest classification approach, we demonstrate the potential to identify individuals who may develop RA, particularly within the 6 to 18-month window before diagnosis.

**HOW THIS STUDY MIGHT AFFECT RESEARCH, PRACTICE OR POLICY**

- Our approach represents a step forward in the development of RA prediction models but also highlights the ongoing difficulty of forecasting disease onset, even in an at-risk cohort. Future research should focus on external validation and inclusion of additional biomarkers to enhance prediction accuracy and clinical applicability.

##  Introduction

Earlier treatment of rheumatoid arthritis (RA) improves long-term outcomes.^1^ Also, individuals at risk of RA are good candidates for preventive interventions.^2^ However, we lack tools to reliably identify individuals with imminent transition towards RA. Indeed, RA results from a complex multi-step process modulated by genetic and environmental risk factors.^3^ Moreover, serum auto-antibodies are not always detectable, especially in case of seronegative RA.^4^

Environmental risk factors increase the likelihood of RA. They include ageing, low alcohol consumption, obesity, female hormonal factors, inhaled pollutants and cigarette smoking.^5^ Genetic components have also been identified in twins and first-degree relatives of RA patients (RA-FDRs).^6^ The so-called ‘shared epitope’ (SE) alleles in the human leukocyte antigen DR beta 1 (HLA-DRB1) genes are the main contributors, but numerous other loci are associated with RA development.^7^ Also, early clinical symptoms identified through the ‘clinically suspected arthralgia’ (CSA) questionnaire have a predictive value for RA onset.^8^ Finally, autoantibodies such as anti-citrullinated protein antibodies (ACPAs) and rheumatoid factors (RFs) develop years before RA with predictive value.^9^

The aim of the present analysis was to develop a prediction model, integrating the various known risk factors using balanced random forests (BRF). The model combines environmental factors, early clinical symptoms, autoantibodies and genetics to identify individuals at high risk of developing RA.

## Methods

### Study population

Patient data came from the prospective cohort SCREEN-RA (www.arthritis-checkup.ch) of RA-FDRs.^10^ Inclusion and exclusion criteria are listed in the supplement.

### Variables

The variables of interest are outlined as follows:

1. Epidemiological and environmental factors, including age, gender, Body Mass Index (BMI), cigarette smoking, occupational dust exposures and diabetes.
2. Serological factors, including established autoantibodies (ACPAs and RFs), and the novel anti-RA33^11^.
3. Clinical symptoms measured using the CSA questionnaire proposed by the European Alliance of Associations for Rheumatology (EULAR). Positivity was defined as satisfying four or more of the seven criteria^8^.
4. Genetic factors, with two variables: the SE and a proprietary Genetic Risk Score (GRS), developed using a neuronal network rule-based algorithm covering 21 independent RA risk variants. Having more than one relative with RA or other autoimmune diseases was also included as a separate predictor, which captures both genetic and environmental risks.
5. A Bernoulli random variable with probability 0.5 was included as a comparison with other variables.

Details on variables can be found in the supplement.

### Outcomes

The primary endpoint was the long-term development of diagnosed RA. The RA diagnosis validation was performed by a board-certified rheumatologist and further validated by the prescription of disease modifying antirheumatic drugs (DMARDs). As a secondary outcome, for exploratory analysis, we considered seropositive inflammatory arthritis (IA) as a surrogate marker for future RA. This was defined as having (1) one or more auto-antibodies associated with RA (ACPA and/or RF positivity (≥1 × manufacturer’s cut-off)) and (2) synovitis on musculoskeletal ultrasound (MSUS) with inflammatory activity, or at least one swollen joint evidenced by physical examination or self-report.^12^ We additionally performed a sensitivity analysis restricted to patients with IA confirmed on clinical examination, excluding those classified based solely on self-reported swelling.

### Statistical analysis

Data were collected, stored and monitored through research electronic data capture (REDCap) software.^13^ Statistical analyses were performed using R (V.4.0.4). Baseline characteristics were compared through Kruskal-Wallis test for continuous variables and Fisher’s exact test for categorical variables.

Time-varying covariates were considered at 3 different timepoints (at the reference date (t0), 6 to 18 months before the reference date (t-1) and 18 months to 36 months before the reference date (t-2)) to assess if predictive value increases closer to diagnosis. The reference date was the last study assessment, and for participants who developed an outcome, either the first date of IA (and when performed, the corresponding MSUS date), or the date of RA diagnosis. Missing values at t0, t-1 and t-2 for each variable are described in online supplemental table S1 and online supplemental table S2.

We chose to use a BRF model because of the highly imbalanced nature of our dataset^14^: the minority class (here, the RA-converters) is much smaller than the majority class (individuals not developing RA). We first split the dataset into five stratified folds to address class imbalance. Within each fold, we further partitioned the training portion into two subsets: one (80%) for model fitting and one (20%) for validation. A BRF was trained on the imputed training subset, with missing values addressed by multiple imputation, using the *mice* package (50 datasets, predictive mean matching, ignoring test rows in the imputation model to avoid data leakage). For each imputed training set, a BRF model was fitted, and predicted probabilities were obtained on the validation subset and on the held-out test subset. Probabilities were then averaged across imputations.

On the validation subset, the classification threshold ensured at least 90% sensitivity while maximising the geometric mean (G-mean) of sensitivity and specificity. This finalised threshold was applied to the test subset for performance assessment. We repeated this process across all five folds, recording sensitivity, specificity, positive predictive value (PPV), negative predictive value (NPV) and G-mean on the test sets. We also computed permutation-based variable importance by measuring the reduction in G-mean following predictor shuffling. Performance metrics and variable importances were averaged across folds. All BRF models were implemented using the ‘randomForestSRC’ package.

## Results

### Characteristics of RA-FDR population at reference date

Of the 1544 participants from the SCREEN-RA cohort, 27 (1.7%) developed RA and 126 (8.2%) developed seropositive IA, during an average follow-up of 7.1 patient-years. Significant differences (p<0.05) between RA and non-RA subgroups were observed for CSA, seropositivity, ACPA, RF, RA33 and the number of multiple RA family members (table 1).

**Table 1:** General characteristics of RA-FDR patients at reference date*

		Non-RA	RA	P value	Non-seropositive IA	Seropositive IA	P value	Missing (%)
n		1517	27		1401†	126‡		
Follow-up time, years (mean (SD))		7.0 (4.5)	4.4 (3.7)	<0.01	6.9 (4.5)	2.4 (2.1)	<0.01	0
Age (mean (SD))		51.2 (14.9)	48.7 (15.9)	0.38	50.3 (14.5)	55.6 (14.5)	<0.01	0
BMI (mean (SD))		24.5 (4.4)	24.1 (3.8)	0.62	24.5 (4.3)	25.4 (5.6)	0.03	20 (1.3)
Gender (%)	Female	1120 (73.8)	24 (88.9)	0.08	1022 (72.9)	108 (85.7)	<0.01	0
More than 1 family member with autoimmune disease (%)	Yes	213 (14.7)	8 (32.0)	0.04	187 (14.0)	29 (24.2)	<0.01	71 (4.6)
Alcohol amount (%)	None	466 (31.9)	7 (26.9)	0.27	432 (32.1)	25 (20.5)	<0.01	58 (3.8)
	Occasionally	564 (38.6)	8 (30.8)		520 (38.6)	51 (41.8)		
	Every week	318 (21.8)	9 (34.6)		302 (22.4)	27 (22.1)		
	Every day	95 (6.5)	1 (3.8)		80 (5.9)	16 (13.1)		
	Several drinks per day	17 (1.2)	1 (3.8)		13 (1.0)	3 (2.5)		
Dust exposure (%)	Yes	97 (7.9)	1 (4.0)	0.71	91 (8.1)	6 (5.2)	0.36	288 (18.7)
Diabetes (%)	Yes	44 (3.3)	2 (9.1)	0.17	41 (3.4)	3 (2.5)	0.79	197 (12.8)
UPA (%)	None	752 (51.7)	15 (55.6)	1.00	696 (51.9)	62 (50.4)	0.25	62 (4.0)
	<10 UPA	265 (18.2)	5 (18.5)		243 (18.1)	22 (17.9)		
	10 to <20 UPA	325 (22.3)	6 (22.2)		302 (22.5)	24 (19.5)		
	20 to <30 UPA	33 (2.3)	0 (0.0)		26 (1.9)	6 (4.9)		
	>=30 UPA	80 (5.5)	1 (3.7)		75 (5.6)	9 (7.3)		
SE (%)	0	781 (52.9)	12 (44.4)	0.59	719 (52.8)	66 (53.2)	0.77	41 (2.7)
	1 copy	584 (39.6)	13 (48.1)		539 (39.6)	51 (41.1)		
	2 copies	111 (7.5)	2 (7.4)		104 (7.6)	7 (5.6)		
Genetic Risk Score (%)	Yes	220 (20.0)	3 (15.0)	0.78	207 (20.7)	13 (11.8)	0.03	422 (27.3)
CSA (%)	Positive	119 (8.8)	11 (42.3)	<0.01	98 (7.9)	35 (28.5)	<0.01	170 (11.0)
Seropositivity (%)	Positive	198 (13.2)	16 (59.3)	<0.01	140 (10.1)	126 (100.0)	<0.01	15 (1.0)
ACPA (%)	Negative	1473 (97.9)	19 (70.4)	<0.01	1365 (98.3)	99 (78.6)	<0.01	12 (0.8)
	Low positivity	18 (1.2)	0 (0.0)		14 (1.0)	17 (13.5)		
	High positivity	14 (0.9)	8 (29.6)		10 (0.7)	10 (7.9)		
RF (%)	Negative	1330 (88.5)	11 (40.7)	<0.01	1267 (91.3)	18 (14.3)	<0.01	15 (1.0)
	Low positivity	85 (5.7)	4 (14.8)		61 (4.4)	67 (53.2)		
	High positivity	87 (5.8)	12 (44.4)		60 (4.3)	41 (32.5)		
RA33 (%)	Negative	657 (92.7)	19 (79.2)	0.05	580 (92.8)	49 (57.6)	<0.01	811 (52.5)
	Low positivity	44 (6.2)	4 (16.7)		37 (5.9)	35 (41.2)		
	High positivity	8 (1.1)	1 (4.2)		8 (1.3)	1 (1.2)		
MSUS (%)		198 (14.1)	6 (22.2)	<0.01	251 (16.5)	57 (45.2)	<0.01	1236 (80.1)
Swollen joints (median (IQR))		0 (0.0–0.0)	0.0 (0.0–1.8)	<0.01	0 (0.0–0.0)	2.0 (1.0–2.0)	<0.01	701 (45.4)

* Reference date was the last study assessment, and for participants who developed an outcome, either the first date of IA (and when performed, the corresponding MSUS date), or the date of RA diagnosis. Data before multiple imputation, NAs not considered. P values computed with Kruskal-Wallis test for continuous variables and Fisher’s exact test for categorical variables. Diabetes: type I & type II. Seropositivity = ‘positive’ if ACPA or RF positive. For ACPA, RF and RA33: ‘low positivity’: (1 to 3 times the manufacturer’s cutoff (MCO) and ‘high positivity’: > 3 × MCO).

† 17 RA-FDRs developed RA and were not included in this control group for analysis.

‡ 9 of these RA-FDRs with IA later developed RA.

ACPA, anti-citrullinated protein autoantibody; BMI, Body Mass Index; CSA, clinically suspected arthralgia=1 if more 4 or more criteria satisfied; IA, inflammatory arthritis; MSUS, musculoskeletal ultrasound; RA33, anti-RA33 autoantibodies; RA, rheumatoid arthritis; RA-FDRs, first-degree relatives of RA patient; RF, rheumatoid factor; SE, shared epitope; UPA, unit-years of pack smoked.

### Balanced random forest (BRF)

Classification results at 6–18 months and 18–36 months yielded moderate G-mean (0.42 to 0.58) for predicting RA and seropositive IA (table 2). Sensitivity remained high (≥0.82), aside from RA at 18–36 months (0.74), whereas specificity was low, reflecting a higher rate of false positives.

**Table 2:** Average fivefold cross-validation results of the balanced random forest at two different time points

	Sensitivity	Specificity	PPV	NPV	G-Mean
Outcome: rheumatoid arthritis					
6 to 18 months before reference date	0.82	0.43	0.03	0.99	0.58
18 to 36 months before reference date	0.74	0.48	0.02	0.99	0.57
Outcome: seropositive inflammatory arthritis					
6 to 18 months before reference date	0.91	0.18	0.09	0.95	0.42
18 to 36 months before reference date	0.88	0.21	0.09	0.95	0.42

Dates: t-1, 6 to 18 months before the reference date and t-2, 18 to 36 months before the reference date.

CV, cross-validation; NPV, negative predictive value; PPV, positive predictive value.

**Figure 1:**
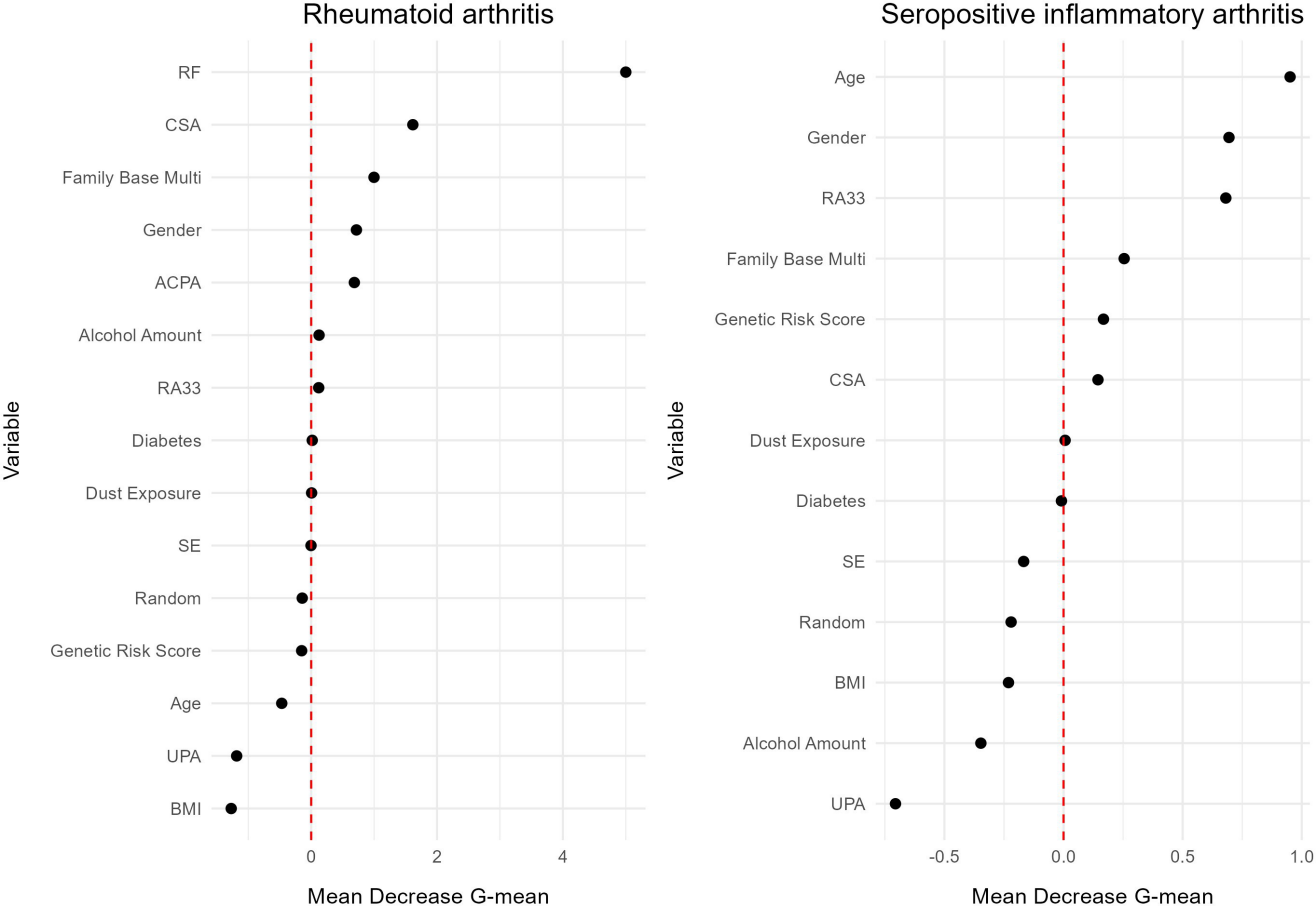
Variable importance for outcomes (i) rheumatoid arthritis and (ii) seropositive inflammatory arthritis, 6 to 18 months before the reference date. Diabetes = type I & type II. Family base multi = more than one first-degree relative has RA and/or another autoimmune disease. Random = random variable from Bernoulli distribution with probability 0.5. ACPA, anti-citrullinated protein autoantibody; BMI, Body Mass Index; CSA, clinically suspected arthralgia; RA, rheumatoid arthritis; RA33, anti-RA33 autoantibodies; RF, rheumatoid factor; SE, shared epitope; UPA, pack-year.

RF had the highest importance in predicting RA, followed by CSA and having multiple diseased family members (figure 1). When predicting seropositive IA, in the absence of ACPA and RF (already defining the outcome), age, gender and anti-RA33 became the most important variables. Model performance and variable importance remained consistent in the analysis restricted to patients with IA confirmed on clinical examination (online supplemental table S3 and figure S4).

## Discussion

We report a multi-factorial approach to identify people at risk of developing RA. As RA has a low incidence in the general population,^15^,^17^ we took advantage of an RA-FDRs cohort with an increased risk of RA. Our approach is novel by including not only epidemiological and serological risk factors but also genetic biomarkers and clinical symptoms.

Analysis of variable importance reaffirmed known RA risk factors: RFs and clinical symptoms (CSA) were the most predictive for RA in at-risk individuals. Surprisingly, some established risk factors, such as ACPA, SE and smoking status, demonstrated no predictive power in our model. This could be due to the small number of RA cases, or the majority of them being ACPA seronegative (70%) at the time of disease onset.

In the absence of RF and ACPA, anti-RA33 and age emerged as significant predictors of seropositive IA in our dataset. For the prediction of IA, the CSA score was no longer a significant predictor, potentially due to the variability and subjectivity in self-reported symptoms and clinical assessments by study nurses. Specifically, joints deformed by erosive osteoarthritis might have been erroneously labelled as swollen. In our cohort, approximately 30% of individuals classified as seropositive IA were included based on self-reported joint swelling, which may have contributed to some heterogeneity in outcome definition. We chose to include self-reported swelling in the seropositive IA definition to capture transient joint swelling, which is frequent during the initial phases of RA development, and maximise the use of available data. Consequently, seropositive IA is less reliable than RA diagnosis confirmed by a rheumatologist, which may explain why the CSA score is not a predictor of seropositive IA in our dataset. The prominence of age likewise reflects joint diseases, including osteoarthritis, in older individuals. Higher age, BMI and alcohol consumption were associated with seropositive IA in this analysis, which may suggest a tendency towards metabolic syndrome, possibly linked to low-grade inflammation or underlying joint disease like osteoarthritis. Future trials in very early RA should probably be careful from distinguishing these cases from true autoimmune synovitis. Further research is needed to clarify these overlaps and prevent misinterpretation.

The model was designed to target a sensitivity of 90% but ultimately achieved around 80%, ensuring that most true positives were identified despite the trade-off in specificity. Clinically, this emphasis on sensitivity is advantageous, as missing fewer cases can lead to earlier interventions and potentially better patient outcomes. Although the resulting specificity remains limited, capturing nearly all high-risk individuals outweighs the drawbacks of false positives in a disease where timely intervention can significantly improve outcomes. Nonetheless, this threshold can be adapted to other performance metrics if different priorities arise. Our approach provides the flexibility, ranging from maximising early detection to minimising overdiagnosis.

A limitation is the small number of incident RA cases in the cohort (27 out of 1544). Furthermore, in an RA-FDR cohort, 1.7% of RA-diagnosed patients is lower than the 5% lifetime prevalence reported in the literature among FDRs. This difference is likely due to a relatively young inclusion age of 43.9 years and a limited follow-up period (around 7 years). Additional RA cases may develop over time, which could affect the relative weight of prognostic factors, such as ACPA. Indeed, 41% of RA converters were seronegative, which is higher than observed in the average Swiss RA patients.^18^ Another limitation is the lack of external validation on a similar cohort. While using RA diagnosis by board-certified rheumatologists for the outcome improves the generalisability of this study, it could be a source of misclassification bias since some patients may not have received a diagnosis.

While our model represents a step forward in RA prediction by combining various risk factors, there is room for improvement. Future research should focus on incorporating larger cohorts, data pooling from multiple sources, adjusting classification thresholds for clinical utility and integrating novel predictors. Such collaborative efforts could provide accurate and sensitive prediction tools, facilitating earlier diagnosis and intervention and improving outcomes for individuals at risk of RA. It is important to emphasise that a high sensitivity model aiming to capture all high-risk individuals carries the risk of overdiagnosis, which may lead to unnecessary anxiety or potentially inappropriate treatments, including DMARDs that can have serious side effects. Therefore, before this model can be implemented in clinical practice, extensive validation and careful calibration of decision thresholds are necessary.

## Supplementary material

10.1136/rmdopen-2025-005773 online supplemental file 1

## Data Availability

Data are available on reasonable request. Anonymised data from the SCREEN-RA cohort can be shared on request (contact senior author Pr. Finckh at axel.finckh@hug.ch).

## References

[1] Burgers LE, Raza K, van der Helm - van Mil AH (2019). Window of opportunity in rheumatoid arthritis – definitions and supporting evidence: from old to new perspectives. RMD Open.

[2] Alpizar-Rodriguez D, Finckh A (2020). Is the prevention of rheumatoid arthritis possible?. Clin Rheumatol.

[3] Deane KD, Demoruelle MK, Kelmenson LB (2017). Genetic and environmental risk factors for rheumatoid arthritis. Best Pract Res Clin Rheumatol.

[4] Ajeganova S, Huizinga TWJ (2015). Seronegative and seropositive RA: alike but different?. Nat Rev Rheumatol.

[5] Petrovská N, Prajzlerová K, Vencovský J (2021). The pre-clinical phase of rheumatoid arthritis: From risk factors to prevention of arthritis. Autoimmun Rev.

[6] Frisell T, Saevarsdottir S, Askling J (2016). Family history of rheumatoid arthritis: an old concept with new developments. Nat Rev Rheumatol.

[7] Okada Y, Wu D, Trynka G (2014). Genetics of rheumatoid arthritis contributes to biology and drug discovery. Nature New Biol.

[8] Burgers LE, Siljehult F, ten Brinck RM (2017). Validation of the EULAR definition of arthralgia suspicious for progression to rheumatoid arthritis. Rheumatology (Oxford).

[9] Wu C-Y, Yang H-Y, Luo S-F (2021). From Rheumatoid Factor to Anti-Citrullinated Protein Antibodies and Anti-Carbamylated Protein Antibodies for Diagnosis and Prognosis Prediction in Patients with Rheumatoid Arthritis. Int J Mol Sci.

[10] Gilbert BTP, Lamacchia C, Mongin D (2021). Cohort profile: SCREEN-RA: design, methods and perspectives of a Swiss cohort study of first-degree relatives of patients with rheumatoid arthritis. BMJ Open.

[11] Sieghart D, Platzer A, Studenic P (2018). Determination of Autoantibody Isotypes Increases the Sensitivity of Serodiagnostics in Rheumatoid Arthritis. Front Immunol.

[12] Brulhart L, Alpízar-Rodríguez D, Nissen MS (2019). Ultrasound is not associated with the presence of systemic autoimmunity or symptoms in individuals at risk for rheumatoid arthritis. RMD Open.

[13] Harris PA, Taylor R, Thielke R (2009). Research electronic data capture (REDCap)—A metadata-driven methodology and workflow process for providing translational research informatics support. J Biomed Inform.

[14] O’Brien R, Ishwaran H (2019). A Random Forests Quantile Classifier for Class Imbalanced Data. Pattern Recognit.

[15] Romão VC, Fonseca JE (2021). Etiology and Risk Factors for Rheumatoid Arthritis: A State-of-the-Art Review. Front Med.

[16] van Boheemen L, van Schaardenburg D (2019). Predicting Rheumatoid Arthritis in At-risk Individuals. Clin Ther.

[17] Karlson EW, van Schaardenburg D, van der Helm-van Mil AH (2016). Strategies to predict rheumatoid arthritis development in at-risk populations. Rheumatology (Oxford).

[18] Gilbert BTP, Mongin D, Aymon R (2024). Comparative effectiveness of baricitinib and alternative biological DMARDs in a Swiss cohort study of patients with RA. BMJ Open.

