# Supplementary material for: Identification of individuals at high risk of developing rheumatoid arthritis: a balanced random forest model in a cohort of 1544 first-degree relatives
Source: RMD Open. 2025 Nov 27;11(4):e005773. doi: 10.1136/rmdopen-2025-005773 (PMC12666099; doi:10.1136/rmdopen-2025-005773)
Supplement: online supplemental file 1 [file rmdopen-11-4-s001.docx]

**SUPPLEMENTARY MATERIALS**

**Genetic variables**

The presence of shared epitope (SE) was determined by HLA genotyping using reverse PCR-sequence-specific oligonucleotide microbead arrays (One Lambda, Canoga Park, CA, USA) and PCR-sequence-specific primers (Genovision, Milan Analytika AG, Switzerland). The method discriminates all the major subtypes in different alleles groups within HLA-DRB1 gene (01:01; 01:02; 03:01 ; 04:01 ; 04:03 ; 04:04 ; 04:05 ; 10:01). PCR-SSP was used to analyze the SE-positive ambiguities in order to obtain the final 4-digit result.

The NGS technology AmpliSeq^TM^ on the Ion GeneStudio^TM^ instruments (Thermo Fisher Scientific, USA) was used covering 320 variants specific for RA. For panel-specific library preparation the Ion AmpliSeq Library Kit v2.0 was used with customized multiplex panel and 100ng gDNA as starting material. The Ion Chef instrument performed the fully automated template preparation and loading of the Chip. For this workflow we used the Ion 530 Chip. The results were analyzed using a newly developed neural network rule-based algorithm containing 14 rules. The genetic approach is part of a patent application (EP 22169795) and information on SNPs and the algorithm can be found after publication of the patent.

**Serological assays**

ACPA positivity was defined by a positive result to at least one of the ACPA assays, according to the manufacturers' cut-off values (anti-CCP2, CCPlus® Immunoscan, Svar Life Science, ≥ 25 U/mL; anti-CCP 3.1, QUANTA Lite® CCP3.1 IgG/IgA, Inova Diagnostics, ≥ 20 U/mL; anti-CCP3, QUANTA Lite® CCP3 IgG, Inova Diagnostics, ≥ 20 U/mL; or EliA™ CCP IgG or IgA assays (Phadia AB, > 10 U/mL). QUANTA Lite RF IgM and IgA® ELISAs (Inova Diagnostics; ≥ 6 U/mL) and EliA RF IgM and IgA (Phadia AB; > 5 IU/mL for IgM and >20 IU/mL for IgA) were used to define RF status. Anti-RA33 positivity was defined by a positive result with at least one of the anti-RA33 prototype assays (IgA, IgG and IgM) using the EliA platform ( Phadia AB, > 12 μg/mL for IgG, > 4.5 μg/mL for IgA and > 32 μg/mL for IgM; cutoff for RA33 isotypes were used as described in Sieghart et al 2018). High positivity was defined by values at least 3 times the manufacturer’s cutoff (MCO).

**CSA**

Clinical symptoms were measured through the ‘Clinically Suspected Arthralgia’ (CSA) questionnaire, composed of seven criteria validated by EULAR:
- Joint symptoms of recent onset (<1 year);

- Symptoms located in MCP joints;

- Duration of morning stiffness >60min;

- Most severe symptoms present in the early morning;

- Being RA-FDR;

- Difficulty with making a fist;

- Positive squeeze test of MCP joints.

CSA positivity was defined as satisfying four or more of the seven criteria.

**Musculoskeletal ultrasound (MSUS) examination**

Significant level of inflammatory activity on articular MSUS or ‘active MSUS’ was defined as a total B-mode score of greater than 8, including at least one joint with significant synovitis (defined as grade 2 or 3) or significant synovial hyperaemia (defined as Doppler score greater than 1).

**Population****, inclusion and exclusion criteria.**

The study population is a genetically defined at risk population, namely FDRs of established RA patients. The study population also comprises a minority of FDRs of patients with lupus or other connective tissue diseases, autoimmune thyroiditis, or type 1 diabetes. Indeed, because of shared genetic risk factors with RA, all these conditions increase the risk of RA among FDRs in a similar magnitude. Other inclusion criteria include being at least 18 years.

Exclusion criteria are an established diagnosis of RA (>12 months), treated with antirheumatic drugs, or the presence of active comorbid inflammatory arthritides (i.e., patients with psoriatic arthritis, spondylarthritis, or known microcrystalline arthritis) to avoid outcome misclassification.

**Table S1**: Number of missing values at the reference date (t0), 6 to 18 months before (t-1) and 18 to 36 months before (t-2) (stratified by RA outcome).

|  | **Non-RA at t0** | **RA at t0** | **Non-RA at t-1** | **RA at t-1** | **Non-RA at t-2** | **RA at t-2** |
| --- | --- | --- | --- | --- | --- | --- |
| n | 1517 | 27 | 1517 | 27 | 1517 | 27 |
| BMI (%) | 20 (1.3) | 0 (0.0) | 20 (1.3) | 0 (0.0) | 20 (1.3) | 0 (0.0) |
| More than 1 family member with autoimmune disease (%) | 69 (4.5) | 2 (7.4) | 69 (4.5) | 2 (7.4) | 69 (4.5) | 2 (7.4) |
| Alcohol amount (%) | 57 (3.8) | 1 (3.7) | 127 (8.4) | 5 (18.5) | 196 (12.9) | 8 (29.6) |
| Dust Exposure (%) | 286 (18.9) | 2 (7.4) | 286 (18.9) | 2 (7.4) | 286 (18.9) | 2 (7.4) |
| Diabetes (%) | 192 (12.7) | 5 (18.5) | 192 (12.7) | 5 (18.5) | 192 (12.7) | 5 (18.5) |
| UPA (%) | 62 (4.1) | 0 (0.0) | 130 (8.6) | 4 (14.8) | 199 (13.1) | 7 (25.9) |
| SE (%) | 41 (2.7) | 0 (0.0) | 41 (2.7) | 0 (0.0) | 41 (2.7) | 0 (0.0) |
| Genetic Risk Score (%) | 415 (27.4) | 7 (25.9) | 415 (27.4) | 7 (25.9) | 415 (27.4) | 7 (25.9) |
| CSA (%) | 169 (11.1) | 1 (3.7) | 257 (16.9) | 4 (14.8) | 257 (16.9) | 8 (29.6) |
| ACPA (%) | 12 (0.8) | 0 (0.0) | 111 (7.3) | 4 (14.8) | 180 (11.9) | 7 (25.9) |
| RF (%) | 15 (1.0) | 0 (0.0) | 113 (7.4) | 4 (14.8) | 181 (11.9) | 7 (25.9) |
| RA33 (%) | 808 (53.3) | 3 (11.1) | 839 (55.3) | 7 (25.9) | 853 (56.2) | 11 (40.7) |

CSA, clinically suspected arthralgia = 1 if more 4 or more criteria satisfied. UPA, pack-year. For ACPA, RF and RA33:” low positivity”: (1 to 3 times the manufacturer’s cut-off (MCO) and “high positivity”: > 3 x MCO). SE, shared epitope. ACPA, anti-citrullinated protein autoantibody. RF, rheumatoid factor. RA33, anti-RA33 autoantibodies. RA, rheumatoid arthritis. t0: reference date, t-1: 6 to 18 months before the reference date, t-2: 18 to 36 months before the reference date.

**Table S2**: Number of missing values at the reference date (t0), 6 to 18 months before (t-1) and 18 to 36 months before (t-2) (stratified by seropositive inflammatory arthritis outcome).

|  | **Secondary Outcome Negative at t0** | **Secondary Outcome Positive at t0** | **Secondary Outcome Negative at t-1** | **Secondary Outcome Positive at t-1** | **Secondary Outcome Negative at t-2** | **Secondary Outcome Positive at t-2** |
| --- | --- | --- | --- | --- | --- | --- |
| n | 1401 | 1401 | 1401 | 1401 | 1401 | 1401 |
| BMI (%) | 9 (1.4) | 1 (0.8) | 19 (1.4) | 1 (0.8) | 19 (1.4) | 1 (0.8) |
| More than 1 family member with autoimmune disease (%) | 63 (4.5) | 6 (4.8) | 63 (4.5) | 6 (4.8) | 63 (4.5) | 6 (4.8) |
| Alcohol amount (%) | 54 (3.9) | 4 (3.2) | 124 (8.9) | 35 (27.8) | 192 (13.7) | 50 (39.7) |
| Dust Exposure (%) | 276 (19.7) | 10 (7.9) | 276 (19.7) | 10 (7.9) | 276 (19.7) | 10 (7.9) |
| Diabetes (%) | 186 (13.3) | 8 (6.3) | 186 (13.3) | 8 (6.3) | 186 (13.3) | 8 (6.3) |
| UPA (%) | 59 (4.2) | 3 (2.4) | 127 (9.1) | 35 (27.8) | 195 (13.9) | 50 (39.7) |
| SE (%) | 39 (2.8) | 2 (1.6) | 39 (2.8) | 2 (1.6) | 39 (2.8) | 2 (1.6) |
| Genetic Risk Score (%) | 400 (28.6) | 16 (12.7) | 400 (28.6) | 16 (12.7) | 400 (28.6) | 16 (12.7) |
| CSA (%) | 153 (10.9) | 3 (2.4) | 244 (17.4) | 36 (28.6) | 248 (17.7) | 50 (39.7) |
| ACPA (%) | 12 (0.9) | 0 (0.0) | 108 (7.7) | 35 (27.8) | 176 (12.6) | 50 (39.7) |
| RF (%) | 13 (0.9) | 0 (0.0) | 108 (7.7) | 35 (27.8) | 176 (12.6) | 50 (39.7) |
| RA33 (%) | 776 (55.4) | 776 (55.4) | 776 (55.4) | 776 (55.4) | 776 (55.4) | 776 (55.4) |

CSA, clinically suspected arthralgia = 1 if more 4 or more criteria satisfied. UPA, Unit-pack-year. For ACPA, RF and RA33: “low positivity”: (1 to 3 times the manufacturer’s cut-off (MCO) and “high positivity”: > 3 x MCO). SE, shared epitope. ACPA, anti-citrullinated protein autoantibody. RF, rheumatoid factor. RA33, anti-RA33 autoantibodies. RA, rheumatoid arthritis. t0: reference date, t-1: 6 to 18 months before the reference date, t-2: 18 to 36 months before the reference date.

**Random Forest Model**

Random forest model grows and combines multiple decision trees and aggregate the result of each decision tree. The term “random” refers to two features of the algorithm: 1) each individual tree is grown using only a random sample of the entire available data, which reduces the risk of overfitting; 2) each “node” of the trees is grown by randomly selecting certain available variables, which makes the trees uncorrelated. It is an “ensemble” technique which combines many weak algorithms (the single decision tree) into a strong one (the random forest).

The code for the full analysis can be found at: <https://gitlab.unige.ch/screen-ra/random_forest>

**Table S3**: Average 5-fold cross-validation results of the balanced random forest at two different time points, with self reported data withdrawn from the analysis (33 patients withdrawn)

|  | **Sensitivity** | **Specificity** | **PPV** | **NPV** | **G-Mean** | |
| --- | --- | --- | --- | --- | --- | --- |
| **Outcome: Seropositive Inflammatory Arthritis** | | | | | |  |
| 6 to 18 months before reference date | 0.84 | 0.30 | 0.08 | 0.96 | 0.42 | |
| 18 to 36 months before reference date | 0.85 | 0.22 | 0.07 | 0.96 | 0.42 | |

CV: cross-validation. PPV, Positive Predictive Value. NPV, Negative Predictive Value. Dates: t-1, 6 to 18 months before the reference date and t-2, 18 to 36 months before the reference date. G-mean =$\sqrt{Sensitivity \times Specificity}$.

**Figure S4**: Variable importance for Seropositive inflammatory arthritis outcome, 6 to 18 months before the reference date, with self-reported data withdrawn from the analysis.


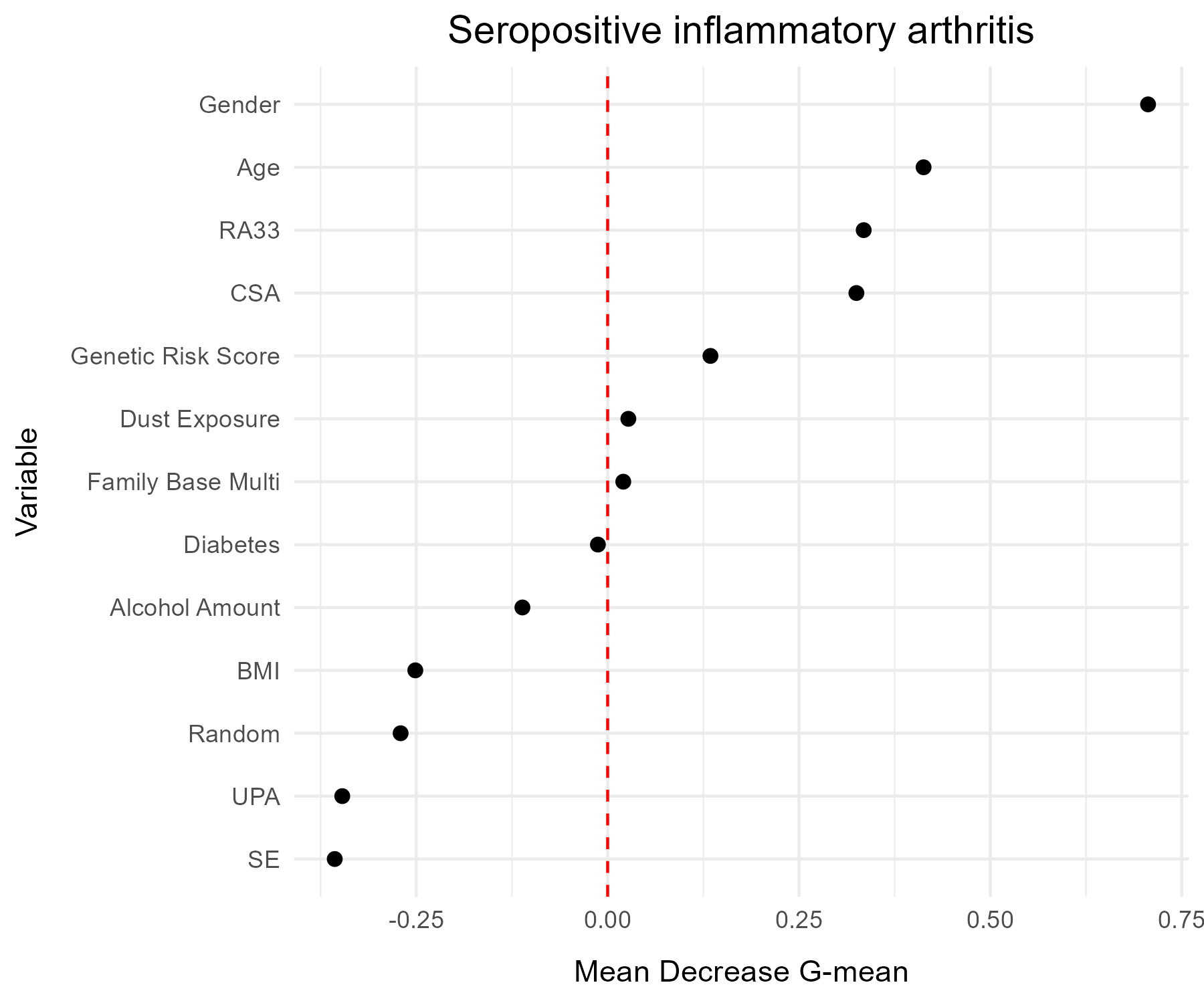


CSA = clinically suspected arthralgia. Diabetes = type I & type II. UPA = pack-year. SE = shared epitope. ACPA = anti-citrullinated protein autoantibody. RF = rheumatoid factor. RA33 = anti-RA33 autoantibodies. RA = rheumatoid arthritis. Family base multi = more than one first degree relative has RA and/or another autoimmune disease. Random = random variable from Bernoulli distribution with probability 0.5.
